# Permeation Protection by Waterproofing Mucosal Membranes

**DOI:** 10.3390/pharmaceutics15122698

**Published:** 2023-11-29

**Authors:** Luisa Coderch, Cristina Alonso, Ana Cristina Calpena, Maria Luisa Pérez-García, Beatriz Clares-Naveros, Anderson Ramos, Meritxell Martí

**Affiliations:** 1 Surfactants and Nanobiotechnology Department, Institute of Advanced Chemical of Catalonia of CSIC (IQAC-CSIC), Jordi Girona 18-26, 08034 Barcelona, Spain; luisa.coderch@iqac.csic.es (L.C.); anderson.ramos@iqac.csic.es (A.R.); meritxell.marti@iqac.csic.es (M.M.); 2 Department de Farmàcia i Tecnologia Farmacèutica, i Fisicoquímica, Facultat de Farmàcia i Ciències de l’Alimentació, Universitat de Barcelona, Avda. Joan XXIII 27-31, 08028 Barcelona, Spain; anacalpena@ub.edu; 3 Institut de Nanociència i Nanotecnologia UB (IN2UB), Universitat de Barcelona, Avda. Joan XXIII 27-31, 08028 Barcelona, Spain; mlperez@ub.edu; 4 Departament de Farmacologia, Toxicologia i Química Terapèutica, Facultat de Farmàcia i Ciències de l’Alimentació, Universitat de Barcelona, Avda. Joan XXIII 27-31, 08028 Barcelona, Spain; 5 Department of Pharmacy and Pharmaceutical Technology, Faculty of Pharmacy, University of Granada, 18071 Granada, Spain; beatrizclares@ugr.es; 6 Biosanitary Research Institute of Granada (ibs GRANADA), Avda de Madrid 15, 18012 Granada, Spain

**Keywords:** mucosa, protection, permeability, kinetic permeation

## Abstract

The permeability of the oral or nasal mucosa is higher than that of the skin. Mucosa permeability depends mainly on the thickness and keratinization degree of the tissues. Their permeability barrier is conditioned by the presence of certain lipids. This work has the main aim of reinforcing the barrier effect of oral mucosa with a series of formulations to reduce permeation. Transmembrane water loss of different formulations was evaluated, and three of them were selected to be tested on the sublingual mucosa permeation of drugs. Caffeine, ibuprofen, dexamethasone, and ivermectin were applied on porcine skin, mucosa, and modified mucosa in order to compare the effectiveness of the formulations. A similar permeation profile was obtained in the different membranes: caffeine > ibuprofen~dexamethasone > ivermectin. The most efficient formulation was a liposomal formulation composed of lipids that are present in the skin stratum corneum. Impermeability provided by this formulation was notable mainly for the low-molecular-weight compounds, decreasing their permeability coefficient by between 40 and 80%. The reinforcement of the barrier function of mucosa provides a reduction or prevention of the permeation of different actives, which could be extrapolated to toxic compounds such as viruses, contaminants, toxins, etc.

## 1. Introduction

The oral mucosa is composed of three layers: the surface layer of oral epithelium, the connective tissue of lamina propria, and the deepest layer of submucosa. The oral epithelium is a squamous stratified layer that covers the entire surface of the oral mucosa. This layer is a highly organized with a maturation pattern similar to that of the skin, with thickness and degree of keratinization changes depending on the location in the oral cavity. This layer acts as a semi-permeable tissue that prevents loss of material from the underlying layer. The oral epithelium acts as a protective barrier against endogenous and exogenous aggression and prevents the permeation of bacterial flora of the oral cavity. Morphological diversity can be found ranging from regions of orthokeratinized mucosa to nonkeratinized mucosa [1]. The nonkeratinized regions of the oral mucosa are more permeable than the keratinized regions, making the floor of the mouth and underside of the tongue more attractive for drug delivery, as well as the buccal regions. In fact, for more than a century, nitroglycerin has been delivered systemically via placement under the tongue to alleviate angina pain [2]. The buccal drug delivery route permits the delivery of much larger molecules than those that can permeate the skin [3].

Permeation across the oral epithelium is mainly conducted via passive diffusion. It is well known that the permeation of actives through the nasal or oral mucosa is greater than that through the keratinized, stratum corneum (SC) tissue of the skin [4]. This main difference in the penetration is due to the different lipid compositions as well as their packing structures. It has been shown that the principal factor of skin barrier function is the lipid content in the stratum corneum. The quantity of corneocyte layers or the epidermal thickness is not expected to play a significant role in the skin function barrier [5,6]. The major lipids present in the skin stratum corneum are ceramides, fatty acids, and cholesterol (Chol), which condition the permeability barrier [7,8]. As stated before, ceramides are one of the main lipids of the skin and play a crucial role in its barrier function [9,10]. In particular, decreased ceramide content can be observed in skin dermatoses [11,12]. Although ceramides alone are not capable of forming liposomal structures [13,14], when they interact with other lipids within the SC lipid matrix, such as, fatty acids, cholesterol, and cholesterol sulfate, they form the stable lipid bilayers [15].

With the aim to strengthen the barrier effect of oral mucosa, waterproof formulations of different hydrophilia with low transmucosal water loss were studied. Much work has been conducted to enhance transmucosal permeation [3,16,17]; however, to our knowledge, no previous works were focused on diminishing mucosal permeation as the present one. At present, semi-synthetic ceramides—in particular, ceramide 3 and ceramide 6—are frequently used in skin care products [18] because of their relatively simple and therefore affordable synthesis. Ceramide treatment is most efficient when the lipids are administered in the form similar to lipid bilayers [13,19,20]. Since the actual lipid matrix of the SC is built on a combination of ceramides, fatty acids, and cholesterol in an almost equimolar ratio [21,22,23], this proportion was mantained in our formulations [24]. Formulations were prepared with Cer 3 and the stratum corneum lipids, as well as with Cer 3 and Cer 6 and the stratum corneum lipids, both at two concentrations of 1% and 10%.

The diffusion resistance of the oral/nasal mucosa is primarily associated with the intercellular lipids of the outer layers of the tissue. Then, the permeability of the oral epithelium is conditioned by the intercellular material. The lipid components of keratinized oral epithelia are similar to those of the epidermis. The major components are neutral lipids formed mainly of ceramides and acyl-ceramides derived from the lamellae of the membrane-coating granules [16]. The epithelium of nonkeratinized oral regions does not contain acylceramides or acylglycosylceramides and small amounts of ceramide but contains relatively high amounts of glycosylceramide [16,25,26,27]. Ceramides are present only in small amounts in nonkeratinized epithelium and there is no mechanism to convert glycosylceramide to ceramide, as occurs in keratinized epithelium [25,26]. Other physiological characteristics that distinguish mucosal tissues from skin, such as an extensive vasculature, their moist surface, and the presence of mucus, should also be taken into account [28,29]. The surface of mucosal membrane is covered with mucus, which contains large glycoproteins (mucins) and is negatively charged. Mucus and saliva have a relevant role in the permeation process, and they are contributing factors in the barrier layer of mucosal tissues [17].

Much work has been conducted on increasing the permeability of mucous membranes to favour the penetration of drugs; however, there is little work aimed at their partial impermeability. Thus, the main objective of the present study is to obtain a formulation that can reinforce the mucosa, increase its barrier effect, and decrease its permeability. This work evaluates the permeability of water and of several drugs (of different physicochemical properties) through different waterproofing formulations. The waterproofing efficacy of these formulations could be the basis to reduce or prevent the penetration of different active ingredients such as viruses, contaminants, toxins, etc.

## 2. Materials and Methods

### 2.1. Materials

Two types of membranes were used: porcine sublingual mucosa as a biological membrane and Whatman^®^ Nuclepore ™ as an artificial membrane, made of polycarbonate and with a pore size of 0.05 µm (Cytiva, Amersham, Buckinghamshire, UK). This artificial membrane has been shown to have a similar permeability to human mucous membranes, and porcine sublingual mucosa [30]. The pig tongues were supplied by the Facultat de Farmàcia i Ciències de l’alimentació of the Universitat de Barcelona from the Hospital de Bellvitge campus with the protocols of the ethics committee and the supervision of said stable. Dermatomed oral mucosa was obtained with a thickness of 500 µm (Dermatome GA630, Aesculap, Tuttlingen, Germany). Therefore, portions of the sublingual oral mucosa were prepared in such a way as to fit the Franz diffusion cells. In addition, to determine the specific thickness, each mucosal portion was measured with a digital micrometer (MAHR, Göttingen, Germany). Porcine skin membrane was supplied by the Department of Cardiology of the Hospital Clínic of Barcelona. Skin from the unboiled back of a Landrace large white pig was dermatomed at 500 ± 50 µm (Dermatome GA630, Aesculap, Germany). Animal handling was approved by the Institutional Review Board and Ethics Committee of Institut d’Investigacions Biomèdiques August Pi i Sunyer (IDIBAPS), and the management of the animals conformed to the Guide for the Care and Use of Laboratory Animals.

Caffeine (CAF), ibuprofen (IBU), dexamethasone (DEX), and ivermectin (IVE) were purchased from Sigma (Sigma-Aldrich, St. Louis, MO, USA). The absorption kinetics of four solutions of these active substances at 1% in MeOH (Merck, Darmstadt, Germany) were tested. The physicochemical properties and biopharmaceutic classification system (BSC) classification of the 4 drugs are detailed in Table 1.

### 2.2. Waterproofing Formulations

The tested formulations fall into three categories: hydrophobic, hydrophilic, and liposomal formulations. Ingredients for the formulations were supplied by Sigma (Sigma-Aldrich, St. Louis, MO, USA), except for the cases provided under below. Water preservation was performed with methylparaben (0.18%), propylparaben (0.02%), and propylene glycol (0.85%). All compounds were purchased from Sigma-Aldrich (St. Louis, MO, USA).

Formulation descriptions are detailed in the following tables: Table 2 for hydrophobic formulations, Table 3 for hydrophilic formulations, and Table 4 for liposomal formulations.

### 2.3. Water Permeability Test

A water permeability test was carried out in order to determine the barrier function of membranes, both artificial (Whatman^®^ Nuclepore ™) and biological (porcine sublingual mucosa). The water permeability parameter was obtained as the transmucosal/transmembranal water loss (TMWL) using a Tewameter TM 300 (Courage-Khazaka, Cologne, Germany).

Transmucosal/transmembranal water loss (TMWL) was measured on the membranes placed in vertical static Franz diffusion cells (3 mL, 1.86 cm^2^, Lara-Spiral, Couternon, France). Franz cells consist of two chambers (donor and receptor chambers) separated by the tested membrane (skin, mucosa or artificial membrane). The receptor chamber was completely filled with receptor fluid, ensuring that it was free of bubbles of air. The receptor fluid was a phosphate saline buffer (pH 7.6) (Sigma-Aldrich, St. Louis, MO, USA) and ethanol (Merck, Darmstadt, Germany) mixed at 1:1 (PBS:EtOH, 1:1). The cells were acclimatized in a thermostatic bath (Julabo, Seelbach, Germany) via continuous stirring of the receptor fluid. After one hour of stabilization, the temperature on the membrane was controlled (32 ± 1 °C) and a first measure of TMWL was made for each membrane. Afterwards, a volume of 70 µL of formulation was applied on the membranes in triplicate and a second measure of TWML was determined at 1 h of this deposition. Additionally, one cell of each type of membrane without any application was used as a control.

### 2.4. In Vitro Release Assay

The in vitro release test was conducted with artificial membrane (Whatman^®^ Nuclepore™) and porcine sublingual mucosa as well as pig skin membrane as a reference. Biological membranes (mucosa and skin) were dermatomed at 500 ± 50 µm of thickness. Membranes were placed in vertical Franz diffusion cells and conditioned in a thermostatic bath at 43 °C to assure a membrane surface temperature of 32 ± 1 °C as in the previous TMWL study. The receptor solution used was the same PBS:EtOH (1:1) as before. Data on the TWML, temperature, and humidity of the membrane were obtained before initiating the test with the Tewameter TM300 (Courage-Khazaka, Cologne, Germany).

On the modified mucosas, a volume of 70 µL of each formulation was applied and after one hour an infinite dose (300 µL) of tested solution was applied. The tested solution was prepared with caffeine (CAF), ibuprofen (IBU), dexamethasone (DEX), and ivermectin (IVE) at 1% in ethanol (Merck, Darmstadt, Germany). Receptor fluid was collected at different times (30 min, 1 h, 2 h, 4 h) and the same volume was immediately replaced with fresh fluid. The aliquots were appropriately diluted and filtered (0.45 µm, Cameo, Sigma-Aldrich, St. Louis, MO, USA) before their analysis with HPLC/DAD.

The release of the active substances was determined with the cumulative amount released (*Qn*, μg/cm^2^), which represents the cumulative amount of the active substances quantified in the receptor fluid per area of the sample [34]. The equation is as follows (1):

(1) $$Qn=\frac{Cn\times Vc+\sum_{i=1}^{n-1}\left(Ci\times Vs\right)}{A},$$

where *Qn* is the cumulative amount of active substance released at time *n* (μg/cm^2^); *Cn* is the concentration of active substance in the collected sample (μg/mL); *Vc* is the volume of the Franz diffusion cell (3 mL); $\sum_{i=1}^{n-1}\;Ci$ is the sum of the active substance concentrations (µg/mL) determined in sampling intervals 1 to *n* − 1; *Vs* is the volume of the collected sample; and *A* is the surface area of the applied membrane (1.86 cm^2^).

The best absorption model to describe the release of active substances was obtained from the graphic representation of data release (values/time), as described by Mallandrich et al. [35]. All data collected were processed over time to obtain the best fit equation for each membrane using the nonlinear regression software STATGRAPHICS^®^ plus 5 (Statgraphics Technologies, Inc., The Plains, VA, USA). Selection of the best equation was made in accordance with the highest correlation coefficient corrected for the number of degrees of freedom (R^2^). Following that analysis, the parameters of the flow (J, µg/mL/h), the permeability coefficient (Kp, cm/h), the delay time (Tl, h), the maximum concentration (Cmax, µg/mL), the maximum time (tmax, h), and the area under the curve (AUC) were calculated for each membrane.

### 2.5. HPLC/DAD Analytical Measurements

The collected samples were analyzed via reverse-phase HPLC, using the Agilent 1620 Infinity II LC System (Waldbronn, Germany) equipped with a quaternary pump (G7111B), autoinjector (G7167A), multicolumn thermostat (G7116A), and WR diode-array detector (G7115A). The software was OpenLab (version 2.2.0). A LiChrocart250-4/LiChrosorb RP-18 (5 µm) column (Merck, Darmstadt, Germany) was used to quantify the active substances, at 25 °C with an injection volume of 40 µL. The elution conditions were methanol/water (Merck, Darmstadt, Germany) at a gradient from 60:40 to 90:10 in 15 min, constant at 90:10 for 15 min and the last 60:40 in 10 min with a flow of 1 mL/min. Detection for caffeine (CAF) was 280 nm, for ibuprofen (IBU) was 221 nm and for dexamethasone (DEX) and ivermectin (IVE) was 240 nm. Methanol (Merck, Darmstadt, Germany) was used as an extraction solvent for all active substances. All analytical procedures were validated following the guidelines developed by the International Conference on Harmonization (ICH) [36]. The calibration curve, limit of quantification (LoQ), and limit of detection (LoD) were obtained and are detailed in Table 5.

### 2.6. Statistical Analysis

Statistical treatment was performed using STATGRAPHICS plus 5 nonlinear regression software (Statgraphics Technologies, Inc., The Plains, VA, USA). The nonparametric Krukall–Wallis test was used due to the abnormal distribution of the data. The permeation parameters of the active substances obtained for each membrane were compared with of porcine skin. A probability level of 0.05 (*p*) was considered statistically significant. All results are expressed as the mean ± standard deviation (SD).

## 3. Results

### 3.1. Water Permeability Test (TMWL)

Screening of 63 formulations was carried out to evaluate the TMWL on the synthetic membrane Nuclepore. The formulations that were found to be more impermeable to this membrane were also evaluated with sublingual mucosa. In Table 6, the results of the water permeability of the artificial membrane and the mucous membrane are indicated.

As seen in the TMWL results, the permeability of the sublingual mucosa (control) was high (72 g/h·m^2^), similar to that of the synthetic membrane (control) (80 g/h·m^2^). Both values are significantly higher than the water permeability of healthy skin, which is usually between 5 and 10 g/h·m^2^ [37]. All formulations increase the barrier function of the membrane. Hydrophobic formulations are those that promote a decrease in permeability to a greater degree, followed by liposomal and hydrophilic formulations.

### 3.2. In Vitro Drug Release Test

An in vitro release test was carried out with vertical diffusion cells with dermatomed porcine skin and sublingual mucosa. The parameters of TMWL, humidity, and surface temperature were evaluated for both the membrane, skin, and mucosa before starting the test. A volume of 70 µL of the optimal formulation (Formulations 3, 6, and 16 according Table 6) was deposited on the sublingual mucosa. After 1 h, the TMWL was remeasured (Table 7), and the values corroborated those obtained previously.

To evaluate the barrier effect of the formulation, the permeation of the four drugs was determined. Even though these active substances are not related to virus and contaminants, they are chosen in order to cover the possible behaviour of other substances such as viruses or toxic substances. The selection of active substances was based on their different physicochemical properties following the biopharmaceutic classification system (BSC). BSC is a biopharmaceutical drug classification scheme for correlating in vitro drug dissolution and in vivo bioavailability. There are four BCS classes depending on the solubility and permeability of the active ingredient properties [38].

**Figure 1:**
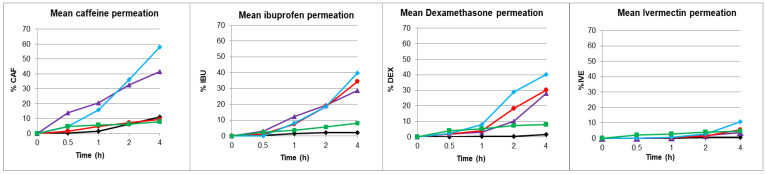
Mean percentage of caffeine (CAF), ibuprofen (IBU), dexamethasone (DEX), and ivermectin (IVE) release applied to the skin (◆), mucosa (⏶), and mucosa modified by formulations F3 (●), F6 (◆), and F16 (■).

Permeation assays were performed in triplicate for the four active substances (CAF, IBU, DEX, and IVE) on porcine skin, sublingual mucosa, and modified sublingual mucosa with F3, F6, and F16. The release of the active pharmaceutical ingredient was determined by the cumulative amount (*Qn*, μg/cm^2^), which corresponded to the total amount of API quantified in the receptor liquid per unit of applied area at the different sampling times. From these values, the rest of the kinetic parameters (such as Flux, J, and C max) were determined as detailed in the experimental section. The percentage and cumulative amount of drug released over time were measured for each of the four active ingredients, obtaining the permeation properties of each ingredient. The results are shown in Figure 1 (exposed to active substances) and expressed in Table 8.

Note the high permeability for caffeine, an intermediate permeability for ibuprofen and dexamethasone, and the extremely low permeability for ivermectin. It can also be noted that while hydrophilic formulation 6 presents permeability values equal to or greater than those of the mucosa, hydrophobic formulation 3, and especially the liposomal formulation 16 decrease permeability, providing a barrier effect similar in some cases to that of the skin.

**Figure 2:**
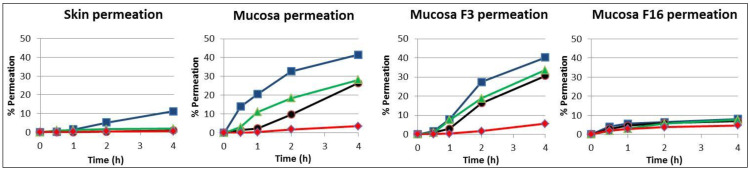
Mean percentage of release of caffeine (■), ibuprofen (⏶), dexametasone (●), and ivermectine (◆) applied to the skin, mucosa, and mucosas modified by formulations F3 and F16.

In Figure 2, the results from the release assay of the four drugs are plotted by type of membrane: skin, unmodified mucosa, and mucosa modified with formulations F3 and F16. The similar profile of the four compounds in skin and mucosa modified with the liposomal formulation F16 is remarkable.

## 4. Discussion

### 4.1. Water Permeability Test (TMWL)

Biological membranes, animal or human, are the gold standard for research on the barrier function of tissue (oral or nasal mucosa). However, due to the high cost and practical difficulties in obtaining these biological membranes as well as preservation, it is necessary to replace them with synthetic membranes with similar properties to those of biological membranes. Moreover, the use of these synthetic membranes would eliminate the previous limitations and will obviate the intra- and intervariability.

This work provides a comparative study between the biological membrane of the sublingual mucosa and an artificial membrane evaluating the barrier function of the membrane. The most widely used test for measuring the integrity of barrier function is the transepidermal water loss [39]. To reinforce the barrier function of both membranes, a first series of 63 formulations was prepared and applied on artificial membranes as well as on sublingual mucosa. The transepidermal water loss (TEWL) or transmembrane water loss (TMWL) was evaluated and those formulations with more favourable barrier protection were selected (Table 6).

Hydrophobic formulations showed a superior barrier effect decreasing the water permeability (TMWL) in both membranes (artificial and biological membranes) after one hour of application. Among them, lipophilic formulations number 3 (F3, Lipophilic Base MI) and number 4 (F4, Lipophilic Base TGC) stand out, in which the value of TWML decreases by more than 90% [32].

Although the distribution of the hydrophilic formulations on the membrane was more uniform and easier to apply than the preceding formulations, water permeability did not decrease in the same order. Formulations number 6 (F6, SCMC gel 4%) and number 9 (F9, PLX-CBP gel) decreased the permeability by between 21 and 24%.

Liposomal formulations have been chosen because their constituents have the ability to structure lipids in aqueous environments. Moreover, formulations F13 to F16 have lipids such as cholesterol, palmitic acid, and ceramides, which are present in the skin and contribute to the protective barrier effect on the skin. The following liposomal formulations can be underlined: formulations 15 (F15) and 16 (F16) with two ceramides, which reduce the water permeability by approximately 40% increasing the barrier effect in both membranes [33].

Since, as indicated in the introduction, mucosal diffusion resistance is mainly associated with lipids in the outer layers of the tissue, a greater effect of hydrophobic formulations, especially on water permeability, would be expected and has been the case.

### 4.2. In Vitro Drug Release Test

To evaluate the changes in permeation through the protected and unprotected membranes to the diffusion of active ingredients, four drugs were tested. In addition, the permeation of the same active substances in porcine skin was studied. As mentioned in the introduction, the barrier structure of mucosa differs greatly from that of the skin. With this in mind, the different formulations were applied to mucous membranes to mimic the skin stratum corneum and contribute to a barrier effect similar to that of the skin. To evaluate the effect of this barrier modification, the permeation of four drugs was determined. The drugs—caffeine (CAF), ibuprofen (IBU), dexamethasone (DEX), and ivermectin (IVE) (Table 1)—were dissolved at 1% in ethanol. The criteria considered in choosing these drugs were the different physicochemical characteristics of solubility and permeability which are directly related to drug absorption and release. Active ingredients belong to a different biopharmaceutical classification system (BCS) group [38]. There are four BCS classes depending on the solubility and permeability of the active ingredient properties. These two properties are crucial in the drug permeation through keratin tissues such as skin or mucous membranes.

Permeation tests were carried out with sublingual mucosa and porcine skin. Once they were conditioned and the TEWL parameter determined, formulations F3, F6, and F16 were applied, and their barrier effect was evaluated (Table 7). Formulation F3 was one that decreased water permeability to a greater degree, as previously observed in Table 6, followed by the liposomal formulation F16 and, to a lesser degree, the hydrophilic formulation F6. The next step was the application of an infinite dose of the drug solution to determine their release parameters. The cumulative percentage of each drug over time was determined (Figure 1). The kinetic parameters of area under the curve (AUC), flux (J), coefficient of permeability (Kp), and maximum concentration (Cmax) were calculated for the 4 active substances (caffeine, ibuprofen, dexamethasone, and ivermectin) (Table 8). The comparison of these parameters for caffeine shows, as expected, an area under the curve, a flux, and a permeability through the unmodified mucosa approximately four times higher than through the skin. Application of the waterproofing formulations indicates that although F6 did not promote mucosal impermeability, both F3 and F16 inferred mucosal impermeability very similar to that of skin. Thus, for a low-molecular-weight hydrophilic compound indicating high permeability such as caffeine, it exhibited skin-like impermeability when F3 and F16 formulations were applied to mucosa, with F16 giving even more impermeable values than skin.

Ibuprofen presented an even more pronounced difference in the area under the curve, flow, and permeability, 14 times higher through the unmodified mucosa compared to the skin. The application of the waterproofing formulations indicates, in this case, that neither F3 nor F6 promoted mucosal impermeability. However, it is worth noting that F16 inferred mucosal impermeability very similar to that of skin. Therefore, for a low-molecular-weight hydrophobic compound with high permeability such as ibuprofen, it exhibits skin-like impermeability when the F16 formulation is applied to the mucosa.

For dexamethasone, it is noteworthy to highlight the pronounced difference in the area under the curve, flow, and permeability between the unmodified mucosa and the skin (50 times higher). No significant changes were observed in the mucosal impermeability when the waterproofing formulations of F3 and F6 were applied. However, it must be noted that the effectiveness of F16, which modified the impermeability of the mucosa, was five times lower than that of virgin mucosa. Therefore, dexamethasone, which is a hydrophilic compound of high molecular weight that indicates low skin permeation, presented a very marked inhibition of its diffusion to mucosa when the F16 formulation was applied, although it did not reach the impermeability of skin.

Ivermectin, as expected, was the active ingredient with the lowest permeability in both skin and mucosa. Moreover, the difference between skin and mucosa was not as marked as for the dexamethasone. In this case, the permeability of ivermectin through the unmodified mucosa was 20 times higher than that through the skin. The application of waterproofing formulations seemed to have less effect. However, it should be noted that F16 inferred an impermeability 1.5 times lower than that of the virgin mucosa to the mucosa without reaching, in any case, the values of the skin. Ivermectin presented a low permeation in skin as a hydrophobic compound with very high molecular weight, but it showed a slight decrease in permeation through mucosa with application of the F16 formulation.

Therefore, it is important to note that the lipophilic formulation F3, which has the greatest effect on water permeation, does not show an increase in its barrier effect to the more hydrophobic compounds. However, the liposomal formulation consisting of the main lipids of the skin substantially increases the barrier effect to all types of compounds tested.

### 4.3. Rank Order Penetration of Drugs

The penetration order of the different compounds through the different membranes always followed a similar profile, with caffeine being the most permeated and ivermectin the least permeated. Ibuprofen and dexamethasone showed a similar intermediate permeation. In all cases, the most waterproofing formulation was F16 composed of lipids that are present in the stratum corneum formed by two types of ceramides structured as liposomes in an aqueous medium. Figure 2 compares the permeation of the four compounds in the porcine skin, unmodified mucosa, and modified mucosa with F3 and F16 formulations. The similar profile of the four compounds in skin and in mucosa modified with F16 is remarkable.

Moreover, the F16 formulation promoted a decrease of approximately 80% in the permeability coefficient of caffeine, ibuprofen, and dexamethasone and approximately 40% for ivermectine, as its high molecular weight favors an ever-decreasing permeation. It is particularly important to highlight the skin-like impermeability provided by this formulation for the compounds with low molecular weights (caffeine and ibuprofen), preventing their penetration. This indicates the preferential effect on potentially more toxic small permeating compounds via the skin and mucosal membrane.

This innovation could, a priori, protect people in general and healthcare workers in particular from being infected by small viruses, such as SARS-CoV-2. Permeation of a virus model with physicochemical properties similar to SARS-CoV-2 has been studied to determine the effect of these formulations [33]. These formulations will probably not provide complete protection; however, the decrease in the permeability coefficient will partially prevent the penetration of the virus at the systemic level.

The projection of the application is directed towards its implementation as a tool in the development of new formulations, new drugs, and in the design and development of drugs or sanitary products suitable to protect from COVID, other viruses, or other toxicants such as biocides.

## 5. Conclusions

The high permeation of actives through the nasal or oral mucosa in contrast to the low penetration through the keratinized stratum corneum of the skin is widely known. This is mainly due to the different lipid compositions and the packing structures they form. Certain lipids determine the permeability barrier. However, they are present only in small amounts in nonkeratinized epithelia. The main objective is to obtain a formulation that can reinforce the mucosa, increase its barrier effect, and reduce or prevent the penetration of different active ingredients such as viruses, contaminants, toxins, etc.

Screening was carried out to evaluate the transmucosal water loss (TMWL) of several formulations on the synthetic membrane and on porcine sublingual mucosa. The kinetic permeation assay was performed for caffeine, ibuprofen, dexamethasone, and ivermectin on porcine skin, mucosa, and modified mucosa after depositing three formulations. The release of the active pharmaceutical ingredient was evaluated through different parameters, and the waterproofing of formulations was determined.

It is worth noting the great permeability of both the artificial membrane (80 g/h·m^2^) and the sublingual mucosa (72 g/h·m^2^) compared to the permeability of the skin (5–10 g/h·m^2^). Hydrophobic formulations are those that decrease permeability to water to a greater degree. However, some hydrophilic formulations were also evaluated for being more palatable. Liposomal formulations were chosen because of their ability to structure lipids in an aqueous environment, particularly with certain lipids that confer a barrier effect. The penetration order of the different compounds in the different membranes always follows a similar profile, caffeine being the most permeable and ivermectin the least permeable, with ibuprofen and dexamethasone demonstrating a similar intermediate permeability. In all cases, the most waterproofing formulation is the one composed of lipids present in the stratum corneum with two types of ceramides structured as liposomes in an aqueous medium. This formulation promotes a decrease of approximately 80% in the permeability coefficient of caffeine, ibuprofen, and dexamethasone and approximately 40% of ivermectin, and its high molecular weight promotes even lower permeability. It is also important to highlight the skin-like impermeability provided by this formulation to the low-molecular-weight compounds (caffeine and ibuprofen), indicating their preferential effect on the possible dermal most toxic compounds through skin and mucosa penetration.

This innovation could, a priori, protect people in general and health care workers in particular from being infected by SARS-CoV-2. These formulations will probably provide limited protection; however, the decrease in permeability will partially prevent the penetration of the virus at the systemic level. This work is directed towards its implementation as a tool in the development of new formulations and new drugs and in the design and development of drugs or sanitary products suitable to protect against COVID-19, other viruses, or other toxicants such as biocides.

## 6. Patents

This work led to three patents:

Alonso, C.; Martí, M.; Coderch, L.; Calpena, A.C.; Mallandrich, M.; Pérez-García, M.L.; Clares, B.; Pérez, N. Lipophilic-based composition. N. Sol: EP23382737.7 (2023) N. Ref: ES1641.1822. CSIC, UB, UGR.

Alonso, C.; Martí, M.; Coderch, L.; Calpena, A.C.; Pérez-García, M.L.; Clares, B. Liposomal-based composition N. de Sol: EP23382651.0 (2023) N. Ref: ES1641.1823. CSIC, UB, UGR.

Alonso, C.; Martí, M.; Coderch, L.; Calpena, A.C.; Pérez-García, M.L.; Giraldo, S.; Bagherpour, S.; Clares, B. Virus model nanoparticle and use thereof. N. de Sol: EP23382736.9 (2023) N. Ref: ES1641.1833. CSIC, UB, UGR.

## Figures and Tables

**Table 1:** Physicochemical properties and BSC classification of active substances evaluated.

Active Substances (API)	pKa	LogP (pH7.4)	MW	BSC Group
Caffeine (CAF)	10.4	−0.07	194.2	1
Ibuprofen (IBU)	5.30	3.97	206.3	2
Dexamethasone (DEX)	12.1	1.74	392.5	1/3 [31]
Ivermectin (IVE)	12.47	5.83	875.1	4

**Table 2:** Description of hydrophobic formulations [32].

Formulation Number	Formulation Name	Composition
1	Tea tree oil mouthwash	Glycerin 15%, sorbitol 4.5%, Lauryl sulfate sodium 3%, ethanol 10% (Merck, Darmstadt, Germany) and Tea tree oil (Acofarma, Terrassa, Spain)
2	Semi-solid anhydrous absorption base	Lecithin 50% in liquid Vaseline
3	Lipophilic base MI	Isopropyl myristate 10% in Filant Vaseline
4	Lipophilic base TGM	Propyl glycol 10%, medium chain triglycerides 10% in Filant Vaseline
5	Fluid anhydrous absorption base	Soy lecithin 50% in Isopropyl palmitate

**Table 3:** Description of hydrophilic formulations.

Formulation Number	Formulation Name	Composition
6	SCMC gel 4%	Sodium carboxymethylcellulose 4%, Glycerin 10% in water
7	SHYL gel 2%	Sodium hyaluronate 2% in water
8	CHIT gel 2%	Chitosan 2%, lactic acid 1% in water
9	ALG gel 4%	Alginate sodium 4%, calcium chloride 4% in water
10	PLX-CBP gel	Poloxamer 26%, Carbopol 940 1% in water

**Table 4:** Description of liposomal formulations [33].

Formulation Number	Formulation Name	Composition
11	PC 10%	Soy Phosphatidylcholine 10% (Lipoid Ludwigshafen, Ludwigshafen am Rhein, Germany)
12	HPC 10%	Soy Hydrogenated Phosphatidylcholine 10% (Lipoid Ludwigshafen, Germany)
13	Cer3 1%	Ceramide3 46.9% (Evonik, Essen, Germany), Cholesterol 30.8%, Palmitic acid 22.4%. Total lipid concentration 1%
14	Cer3 10%	Ceramide3 46.8% (Evonik, Essen, Germany), Cholesterol 31.6%, Palmitic acid 23.0%. Total lipid concentration 10%
15	Cer3Cer6 1%	Ceramide3 24.6% (Evonik, Essen, Germany), Ceramide6 26.5% (Evonik, Essen, Germany), Cholesterol 35.5%, Palmitic acid 22.8%. Total lipid concentration 1%
16	Cer3Cer6 10%	Ceramide3 23.7% (Evonik, Essen, Germany), Ceramide6 24.0% (Evonik, Essen, Germany), Cholesterol 31.6%, Palmitic acid 23.0%. Total lipid concentration 10%

**Table 5:** Linear equation and limit parameters of quantification (LoQ) and detection (LoD) from the HPLC/DAD analysis for caffeine, ibuprofen, dexamethasone and ivermectin.

	**Caffeine (CAF)**	Ibuprofen (IBU)	Dexamethasone (DEX)	Ivermectin (IVE)
Linear Reg. Eq. (R^2^)	$A=51.512\left[CAF\right]+2.002$ (0.9993)	A=58.407[IBU]−8.753 (0.9996)	*A* = $51.352\left[DS\right]+2.745$ (0.9999)	A=42.000[IVE]−4.005 (0.9996)
LoD/LoQ (µg/mL)	0.82/2.49	0.50/1.52	0.23/0.70	0.55/1.66

**Table 6:** Transmembrane water loss (TMWL) of the artificial membrane, the porcine sublingual mucosa, and these membranes modified via the application of the different formulations.

Formulations	Nuclepore TMWL 1 h (g/h·m^2^)	Sublingual Mucosa TMWL 1 h (g/h·m^2^)
Nuclepore control	80.8	--
Sublingual mucosa control	--	72.4
Hydrophobic formulations		
F1 Tea tree mouthwash	71.3	58.5
F2 Semi-solid anhydrous absorption base	15.98	23.6
F3 Lipophilic base MI	2.6	6.5
F4 Lipophilic base TGCM	3.6	3.0
F5 Fluid anhydrous absorption base	22.5	34.2
Hydrophilic formulations		
F6 SCMC gel 4%	64.6	53.8
F7 SHYL gel 2%	75.4	59.3
F8 CHIT gel 2%	75.2	63.0
F9 ALG gel 4%	74.5	62.9
F10 PLX-CBP gel	74.8	57.0
Liposomal formulations		
F11 PC 10%	57.8	66.1
F12 HPC 10%	68.0	63.7
F13 Cer3 1%	59.3	65.6
F14 Cer3 10%	57.2	60.8
F15 Cer3Cer6 1%	49.2	47.2
F16 Cer3Cer6 10%	50.4	45.1

**Table 7:** Application of formulations 3, 6, and 16 and evaluation of the transmucosal water loss, of the skin, of the porcine sublingual mucosa, and of the same modified via the application of the different formulations before applying the drugs.

Sample	Amount Applied	TMWL (g/m^2^/h)
Skin	---	13.68 ± 1.22
Sublingual mucosa	---	84.72 ± 4.44
Sublingual mucosa + F3 (Lipophilic base MI)	51.87 mg	4.17 ± 1.73
Sublingual mucosa + F6 (SCMC gel 4%)	70 µL	57.63 ± 4.72
Sublingual mucosa + F16 (Cer3Cer6 10%)	70 µL	45.05 ± 2.35

**Table 8:** Mean values of area under the curve (AUC), flux (J), coefficient of permeability (Kp), and maximum concentration (Cmax) for active ingredients through the skin, sublingual mucosa, and sublingual mucosa with the protective waterproofing formulations.

API	Parameter	SKIN	MUCOSA	MUCOSA F3	MUCOSA F6	MUCOSA F16
CAF	AUC ((mg/cm^2^)·h)	0.41 ± 0.27	2.19 ± 0.93	0.49 ± 0.13	2.51 ± 0.58	0.45 ± 0.09
	Flux. J (µg/cm^2^/h)	75.09 ± 51.32	428.40 ± 81.41	102.73 ± 30.73	611.08 ± 85.12	78.93 ± 11.05
	Permeability Coef. Kp (10^−3^ cm/h)	6.06 ± 4.12	34.55 ± 0.01	8.28 ± 2.48	49.28 ± 0.01	6.37 ± 0.89
	Lag Time. TL (h)	0.65 ± 0.24	0.04 ± 0.34	0.10 ± 0.08	0.28 ± 0.08	−0.18 ± 0.03
	Maximal Conc. Cmax (µg/mL)	107.23 ± 57.73	514.46 ± 119.97	118.25 ± 34.84	719.67 ± 92.99	99.33 ± 8.49
IBU	AUC ((mg/cm^2^)·h)	0.10 ± 0.01	1.27 ± 0.49	1.32 ± 0.16	1.68 ± 0.47	0.37 ± 0.0002
	Flux. J (µg/cm^2^/h)	24.79 ± 2.75	346.91± 5.87	498.25 ± 49.45	451.01 ± 98.04	74.41 ± 0.16
	Permeability Coef. Kp (10^−3^ cm/h)	2.05 ± 0.23	28.67 ± 0.49	41.18 ± 4.09	37.27 ± 8.10	6.15 ± 0.001
	Lag Time. TL (h)	−0.24 ± 0.19	0.17 ± 0.07	0.23 ± 0.01	0.35 ± 0.06	0.15 ± 0.003
	Maximal Conc. Cmax (µg/mL)	21.93 ± 1.98	340.76 ± 63.62	406.04 ± 36.48	545.68 ± 114.46	96.52 ± 0.08
DEX	AUC ((mg/cm^2^)·h)	0.03 ± 0.01	0.83 ± 0.19	1.10 ± 0.18	1.38 ± 0.55	0.38 ± 0.06
	Flux. J (µg/cm^2^/h)	5.84 ± 3.46	335.25 ± 61.25	330.43 ± 11.68	289.38 ± 99.51	68.26 ± 1.67
	Permeability Coef. Kp (10^−3^ cm/h)	0.49 ± 0.29	28.17 ± 5.15	27.77 ± 0.98	24.32 ± 8.36	5.74 ± 0.14
	Lag Time. TL (h)	0.62 ± 0.16	0.31 ± 0.13	0.36 ± 0.04	0.64 ± 0.36	−0.11 ± 0.27
	Maximal Conc. Cmax (µg/mL)	67.41 ± 95.05	315.34 ± 87.38	363.51 ± 41.15	493.36 ± 131.99	82.14 ± 9.84
IVE	AUC ((mg/cm^2^)·h)	0.026 ± 0.002	0.13 ± 0.02	0.17 ± 0.01	0.31 ± 0.24	0.27 ± 0.24
	Flux. J (µg/cm^2^/h)	3.13 ± 0.86	63.75 ± 1.57	87.23 ± 21.83	144.86 ± 29.93	41.70 ± 5.67
	Permeability Coef. Kp (10^−3^ cm/h)	0.25 ± 0.07	5.06± 0.13	6.92 ± 1.73	11.50 ± 2.38	3.17 ± 1.62
	Lag Time. TL (h)	0.59 ± 0.28	0.24 ± 0.001	0.28 ± 0.02	0.32 ± 0.10	−0.09 ± 0.46
	Maximal Conc. Cmax (µg/mL)	7.38 ± 0.13	44.45 ± 1.68	69.66 ± 18.61	134.92 ± 79.36	59.13 ± 40.84

## Data Availability

Data are contained within the article.
